# An unusual case of leg wound made by a Sea Shell (Scapharca inaequivalis)

**DOI:** 10.1016/j.ijscr.2020.01.039

**Published:** 2020-02-06

**Authors:** Virginia Marina, Florina Popa

**Affiliations:** a Medical Department of Occupational Health, “Dunarea de Jos” University of Galati, Faculty of Medicine and Pharmacy, Galati, Romania; b Department of General Surgery, “Dunarea de Jos” University of Galati, Faculty of Medicine and Pharmacy, Galati, Romania

**Keywords:** ER, emergency room, Shell, Leg injury, Scapharca inaequivalis, Foreign body

## Abstract

**Introduction:**

Most of beaches tend to have a lot of shells and other sharp objects that might hurt you while you’re walking on the beach. Cuts, particularly on the bottom of feet, are common at the beach. In this article we want to present a rare case of a little girl with a knee wound made by a seashell, while playing in the water.

**Case presentation:**

A 9-year-old female patient was presented to the ER with a left knee injury because of a penetrating Scapharca inaequivalis. Since this type of trauma is considered a surgical emergency, taking a thorough history along with careful examination to find out the mechanism and cause of the trauma is crucial towards correct diagnosis and management of the disease. The patient underwent a local surgical procedure to remove the foreign body. Interestingly, the seashell (Scapharca inaequivalis) pinned her left knee in water.

**Conclusions:**

Through this article we want to draw attention to the puncture wounds and cuts caused by sea shells. The particuliarity lies in the fact that the sea shells injuries can be made both in water and on the sand. One main concern with cuts on the feet is risk of infection, especially with diabetic patients.

## Highlights

• Most of beaches tend to have a lot of shells and other sharp objects that might hurt you while you’re walking on the beach.
• A 9-year-old female patient was presented to the ER with a left knee injury because of a penetrating Scapharca inaequivalis.
• The patient underwent a local surgical procedure to remove the foreign body. Interestingly, the seashell (Scapharca inaequivalis) pinned her left knee in water.
• Through this article we want to draw attention to the puncture wounds and cuts caused by sea shells.
• The particuliarity lies in the fact that the sea shells injuries can be made both in water and on the sand.

## 1 Background

The leisure time increases during holidays lead to a higher involvement in beach activities, especially during the summer. Many tourists come from places where there are no beaches or because the characteristics of the beaches are different, before planning their trip they should be informed about the risks they might encounter going on the beach. There is a variety of common foot injuries and conditions that affect individuals especially when walking on the beach. One main concern regarding cuts on the feet is risk of infection, especially in diabetic patients.

One of the key means to diminish the risk of accidents at beaches is to take into account the environment in which they happen: aquatic environment (where jellyfish or other dangerous marine creatures are found) or sandy beach (where sharp foreign objects, glass shards, sea shells can be mixed or hidden in the sand).

Some species of jellyfish may cause local and also systemic reactions. Two members of the scyphozoan phylum that have made dramatic appearances in the Black Sea are Rhizostoma pulmo and Aurelia aurita.

Constanta beach, situated on the Romanian Black Sea coast, is one of the best beaches in Europe visited by many tourists every year. There are shells on some parts of the beach and pure Black Sea creamy coloured sand in other parts. There are various species of Mollusca from Venus gallina and Donax trunculus to the Heavy shells of Scapharca inaequivalis living on the shallow sandy bottom along the Black Sea coast. There are tiny transparent shells of Lucinella divaricata and Lentidium mediterraneum everywhere on the sandy beach.

Many different pathologies can be seen in the ER (Emergency Room) and doctors should be competent in establishing the diagnosis and treatment of all type of wounds [1]. One main concern regarding cuts on the feet is risk of infection, especially in diabetic patients. The work has been reported in line with the SCARE [2].

## 2 Case presentation

**Figs. 1 and 2:**
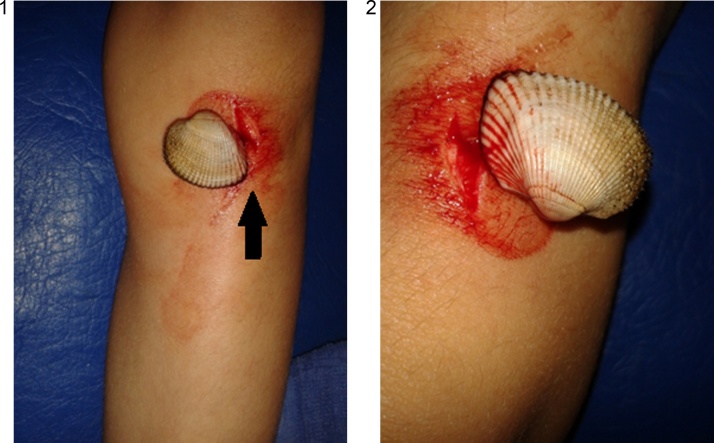
Leg injury made by the seashell.

An 9-year-old female patient was admitted to the emergency medical center accusing pain and the presence of a foreign body in the knee region. According to the patient and her family, the little girl was in the Black sea water when suddenly she felt pain and numbness in the region of the left knee. Her parents helped her to get out of the water and brought her on the beach. At that moment they noticed the presence of a sea shell pinned in the left knee region (Figs. 1 and 2). They tried to pull it out without any success. The sea shell was stuck in the girl’s knee and the more the parents insisted on pulling it out, more pain the little girl felt.

**Fig. 3:**
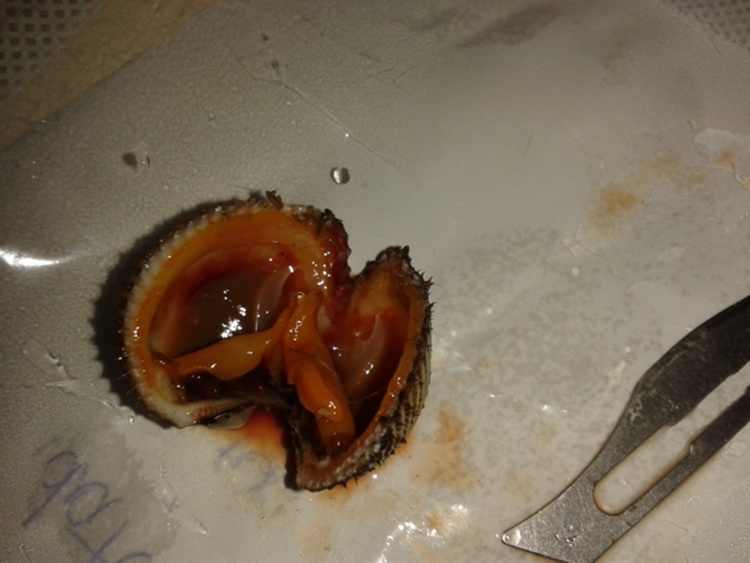
The seashell (Scapharca inaequivalis) excised from the knee region.

A local anesthetic was applied and the sea shell was removed by making an incision and extracting it (Fig. 3). After disinfecting the wound a suture was made to close it. A bandage was applied to protect the wound and the patient received a tetanus shot. The patient was discharged from hospital immediately after with a good general condition and all the necessary recommendations.

## 3 Discussion

People go to the beach to have fun and enjoy themselves, to relax, but they are exposed to several hazards. Some of them can be avoided, but some cannot (our case).

Rhizostoma pulmo can sting your skin with its thread-cells located on the lacy peristomal tentacles. It is better to avoid it when swimming. Moon jellyfish Aurelia aurita is another Scyphozoan medusa common in the Black Sea. Its thread-cells located on the canopy fringe tentacles are less potent weapon than those of Rhizostoma. Contact of this jellyfish with eyes still should be avoided. Jellyfish dermatitis should not be underestimated. Skin reactions can also be accompanied by complex systemic toxic symptoms which are a challenge for physicians.

Sea shells are sharp and brittle, often leaving embedded foreign bodies in deeper wounds. Special care to debridement, wound cleansing, and foreign body removal can help prevent serious wound infections [3]. Foreign bodies may not be symptomatic until the secondary inflammatory reaction occurs 2–3 weeks later.

In our case, diagnosis of the foreign body was easily established because the seashell remained attached to the surface of the skin. Commonly observed complications after hardware removal are infections, impaired wound healing, refractures, tissue and nerve damage and post-operative bleeding or an incomplete removal. Our patient presented no trauma history, that is why the wound did not develop complications and it healed quickly.

Superficial cuts which do not show signs of infection such as swelling, redness, or oozing, can be treated by applying a topical antiseptic and bandage. Adequate source control is mandatory for ER, and early surgical intervention should be considered whenever the patient does not respond to broad-spectrum antibiotic treatment.

Another important issue to discuss is referring to diabetic patients who can face serious foot safety risks on the beach. Diabetes causes poor blood circulation and numbness in the feet. A diabetic may not feel pain from a cut, puncture wound or burn. Any type of skin break on a diabetic foot can easily get infected and ulcerate if it isn’t noticed immediately. Considering these aspects we recommend diabetics wear footwear on the beach, and take them off regularly to check for foreign objects like sand and shells that can cause their feet sores, ulcers and infections [4].

The particularity of this case lies in the fact that the injury occurred in the water, the sea shell got stuck to the foot while the girl was playing into the water. There is lack of information regarding such kind of injuries (only 3 articles published on Pubmed referring to beach-related injuries, one of which dates from 1997). More information as a result of more investigations for such kind of beach injuries will help people and doctors to identify independent risk factors in a beach environment.

## 4 Conclusion

Injuries that can happen in a beach should not be ignored as they can lead to serious health problems. It is important, especially during the summer season, that people should be aware about risk factors for injuries in a beach environment. Knowledge about these conditions and their treatment improves the safety, comfort, and educate people how to stay safe on the beach.

## Sources of funding

No funding for the research.

## Ethical approval

This is not a research study. No ethical approval was necessary.

## Consent

Consent for publication was obtained from the parents of the patient being a minor.

## Author contribution

Popa Florina – wrote the article, processed the pictures.

Marina Virginia –collected the data, took the pictures.

## Registration of research studies

This is a case report. Is not a research study.

## Guarantor

Popa Florina.

## Provenance and peer review

Not commissioned, externally peer-reviewed.

## Declaration of Competing Interest

The authors declare that there is no conflict of interest regarding the publication of this article.
